# Influence of Ambient Temperature on Resting Energy Expenditure in Metabolically Healthy Males and Females

**DOI:** 10.1016/j.tjnut.2025.01.013

**Published:** 2025-01-13

**Authors:** Sara Henkel, Petra Frings-Meuthen, Christina Diekmann, Martin Coenen, Birgit Stoffel-Wagner, Robert Németh, Dominik Pesta, Sarah Egert

**Affiliations:** 1 Institute of Nutritional and Food Sciences, University of Bonn, Bonn, Germany; 2 German Aerospace Center, Institute of Aerospace Medicine, Cologne, Germany; 3 Institute of Clinical Chemistry and Pharmacology, University Hospital Bonn, Bonn, Germany; 4 Institute of Medical Biometry, Informatics and Epidemiology (IMBIE), University Hospital Bonn, Bonn, Germany; 5 Medical Faculty, University of Cologne, Cologne, Germany; 6 Center for Endocrinology, Diabetes and Preventive Medicine (CEDP), University Hospital Cologne, Cologne, Germany; 7 Cologne Excellence Cluster on Cellular Stress Responses in Aging-Associated Diseases (CECAD), University of Cologne, Cologne, Germany

**Keywords:** ambient temperature, resting energy expenditure, REE, resting metabolic rate, RMR, heart rate, heart frequency, body core temperature, heat, mild cold

## Abstract

**Background:**

It is not yet clear to what extent the physiologic regulatory mechanisms that maintain core body temperature are reflected by changes in resting energy expenditure (REE). Particularly, in indirect calorimetry with a canopy, the effects of short-term temperature exposures have not yet been investigated. This can be of relevance for the determination of REE in practice.

**Objectives:**

This randomized crossover study investigated the influence of a 30-min exposure to 18 °C (cool room temperature), 22 °C (room temperature), 28 °C (thermoneutral zone), and 38 °C (heat) on REE determined by assessing the inhaled oxygen volume and the exhaled carbon dioxide volume via indirect calorimetry on 4 consecutive days.

**Methods:**

In total, 32 metabolically healthy participants [16 males and 16 females, age: 25 ± 3 y, body mass index (BMI): 22.4 ± 1.6 kg/m^2^] were included in the study after screening examination.

**Results:**

Lean body mass, ambient temperature, and heart rate were the most important determinants (all *P* < 0.001) and explained 61.3% of the variance in REE. A multivariate linear mixed model analysis revealed that lean mass (15.87 ± 3.66; *P* < 0.001) and ambient temperature (*P* = 0.001) significantly influenced REE. REE significantly differed between 18 and 28 °C (18 °C: +96 ± 24 kcal/24 h; *P* < 0.001), 22 and 28 °C (22 °C: +73 ± 24 kcal/24 h; *P* = 0.003), and 18 and 38 °C (18 °C: +57 ± 23 kcal/24 h; *P* = 0.016).

**Conclusions:**

Effects of ambient temperature on REE, especially cold, are detectable after only brief exposure, emphasizing the importance of performing indirect calorimetry with a canopy under controlled environmental conditions.

The study was registered at clinicaltrials.gov as NCT05505240 (Influence of Ambient Temperature on Resting Energy Expenditure of Healthy Adults - Full Text View - ClinicalTrials.gov).

## Introduction

Determination of resting energy expenditure (REE) forms the basis for the calculation of total energy expenditure (TEE) of an individual (in kilocalories or megajoules per 24 h). REE makes up the largest part (∼50%–70%) of TEE and can be determined by respiratory gas analysis according to the principle of indirect calorimetry, for example, by the canopy method [1]. REE mainly depends on the amount of lean body mass (LBM), as well as age, sex and total body mass, and the influence of these factors can be primarily explained by associated changes in the proportion of LBM [2].

Previous studies provide evidence for changes in REE upon various temperature exposures over a prolonged period [[3], [4], [5], [6], [7], [8], [9], [10], [11], [12], [13], [14]]. Most of these studies were performed in respiration chambers over an interval of ≥12 h to investigate the influence on TEE [4,5,[8], [9], [10], [11], [12], [13], [14]]. The temperature interventions were between 16 and 22 °C as a cold intervention and between 22 and 28 °C as baseline [4,5,[9], [10], [11], [12], [13], [14]]. Compared with the baseline, a cooler ambient temperature significantly increased TEE [4,5,[9], [10], [11], [12], [13], [14]]. Langeveld et al. [7] examined REE of 10 healthy adults at 18 and 24 °C for 2.5 h via indirect calorimetry using a canopy and reported that REE increased significantly at 18 °C. There is little evidence regarding the influence of heat on REE. Valencia et al. [8] investigated the influence of 24-h exposure to 4 ambient temperatures (20, 23, 26, and 30 °C) on TEE in a respiration chamber. TEE was significantly lower at 23 and 26 °C than that at 20 and 30 °C. In their intervention studies, Banerjee and Saha [3] and Gold et al. [6] found that REE was significantly higher at a temperature of 38 °C or higher than that at lower temperatures during the execution of different activities. A field study by Frings-Meuthen et al. [15] also provides evidence that ambient temperature can influence REE. A multivariate model showed a significant positive correlation between ambient temperature and REE at ambient temperatures between 24 and 33 °C [15]. Henderson et al. [16] investigated the effect of heat stress on multiple physiologic variables in a controlled crossover trial. REE increased by 35% upon exposure to 40 °C at 25% relative humidity compared with baseline and by a further 13% upon exposure to 50 °C at 50% relative humidity.

Previous studies indicate that REE changes upon longer-term exposure to different temperatures. However, it is unclear as to what extent the physiologic regulatory mechanisms that maintain core body temperature are reflected by changes in REE after short-term exposures to different ambient temperatures. The recommended ambient temperature for performing indirect calorimetry using a canopy is 20–25 °C with light clothing [17]. However, there is no scientific evidence for use of this temperature range for a standard measuring period (∼20–60 min), as all related studies assessed the influence of ambient temperature over a longer period. This raises the questions of whether the physiologic mechanisms for adapting core body temperature to different ambient temperatures are already reflected in REE after a short period and how large the actual deviations in REE are at temperatures lower or higher than the recommended temperature range of 20–25 °C.

This study was designed to investigate the influence of ambient temperatures of 18 °C (cool room temperature), 22 °C (room temperature), 28 °C (corresponding to the thermoneutral zone of most people), and 38 °C (heat) on the inhaled oxygen volume (VO_2_) and the exhaled carbon dioxide volume (VCO_2_) and the resulting REE under otherwise standardized conditions. We hypothesized that the regulatory mechanisms for heat production and heat dissipation increase REE. Therefore, it was assumed that REE should be higher at 18 and 38 °C than that at 22 and 28 °C. Furthermore, it was assumed that REE should be the lowest at 28 °C among the various intervention temperatures.

## Methods

### Participants

**FIGURE 1:**
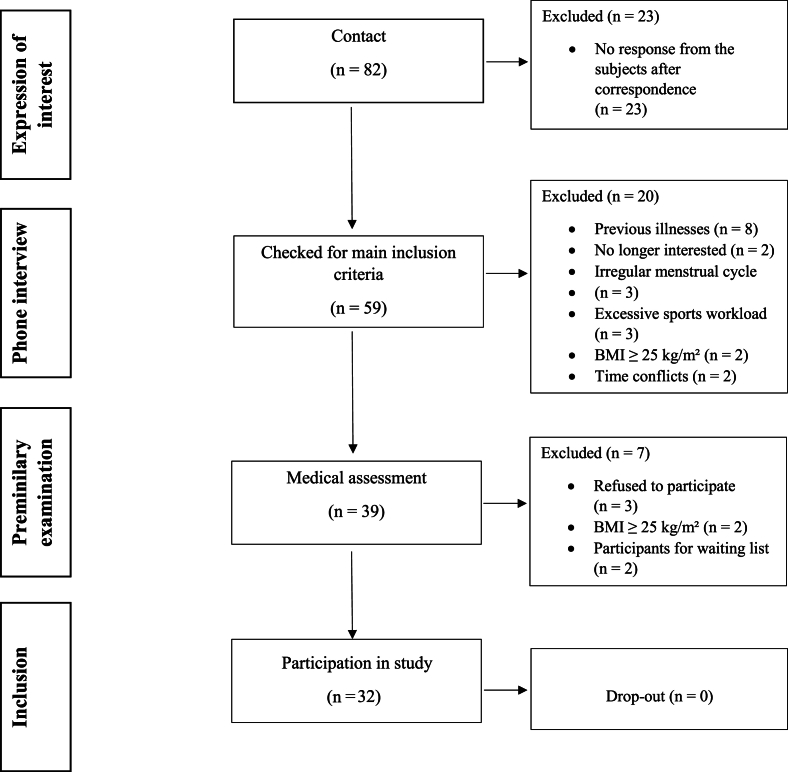
Flowchart of participant recruitment.

Participants were recruited from the University of Bonn by flyers and via an announcement on the homepage of the Institute of Aerospace Medicine of the German Aerospace Center, Cologne, Germany. Of 82 interested subjects, 59 participated in a telephone anamnesis (including medical history and dietary habits) and 39 attended a screening to assess study eligibility (Figure 1). The screening session comprised fasting blood sampling (analyzed for liver, kidney, and thyroid functions, serum lipids and lipoproteins, plasma glucose, and hematology) and physical assessments [determination of REE (Quark-RMR; COSMED), body height and mass, waist and hip circumference, fat mass (FM) and fat-free mass (FFM) (BODPOD system; COSMED), resting blood pressure (BP) and heart rate (HR) (Boso Carat Professional; Bosch + Sohn GmbH)]. Inclusion criteria were age of 18–35 y, normal weight (BMI: 18.5–24.9 kg/m^2^) with weight stability (±3 kg in last 6 mo), nonsmoking, and good metabolic health (based on liver and kidney functions, serum lipids, glucose, hematology, and thyroid function according to the benchmarks for healthy adults). Thirty-two subjects (16 males and 16 females) participated in the study. There was no dropout during the study, and therefore, 32 subjects were included in the analysis. Their baseline characteristics are presented in Table 1.

**TABLE 1:** Baseline characteristics of the subjects (*N* = 32).

	Males (*n* = 16)		Females (*n* = 16)		Total (*N* = 32)	
	Mean	SD	Mean	SD	Mean	SD
Age (y)	25.8	3.3	23.9	2.6	24.9	3.1
Body height (cm)	177.0	5.5	171.5	6.5	174.3^1^	6.5
Body weight (kg)	71.0	8.5	64.5	6.3	67.8^1^	8.1
BMI (kg/m^2^)	22.7	1.7	22.1	1.5	22.4	1.6
FFM (kg)	59.7	6.9	47.2	3.9	53.5^2^	8.4
FM (kg)	11.3	5.9	17.3	3.6	14.3^3^	5.7
FM (%)	15.9	8.3	26.8	5.6	21.1^3^	8.4
Resting systolic blood pressure (mm Hg)	121	19	113	7	117^1^	14
Resting diastolic blood pressure (mm Hg)	76	10	73	6	75^1^	8
Resting pulse rate (bpm)	64	16	64	11	64	14
REE (kcal/24 h)^4^	1824	157	1573	125	1698^2^	189

*Abbreviations:* FM, fat mass; FFM, fat-free mass; REE, resting energy expenditure.

1 Differences between males and females: *P* < 0.05 (Mann–Whitney *U* test).

2 Differences between males and females: *P* < 0.001 (Mann–Whitney *U* test).

3 Differences between males and females: *P* < 0.01 (Mann–Whitney *U* test).

4 Measured at room temperature (∼22 °C, subjects were wearing long-sleeved clothes).

The study was conducted according to the guidelines of the Declaration of Helsinki and all procedures involving human subjects were approved by the ethical committee of the Medical Faculty of the University of Bonn, Germany (approval number 313/22). Written informed consent was obtained from all subjects. The study was registered at clinicaltrials.gov” (NCT05505240).

### Study design

The study was conducted between November 2022 and April 2023 using a randomized crossover design. Subjects came to the study center (German Aerospace Center, Institute of Aerospace Medicine, Cologne, Germany) on 4 consecutive days at the same time in the morning. The intervention temperatures were 18 °C (cold room temperature), 22 °C (room temperature), 28 °C (thermoneutral zone), and 38 °C (heat). Subjects were exposed to these temperatures in a randomized order, with 1 intervention temperature applied per day. Interventions were conducted in a temperature-controlled study room equipped with an air conditioning system. Ambient temperature, air pressure, and air humidity were permanently monitored and recorded by a data logger (ALMEMO 809 V7; Ahlborn Mess- und Regelungstechnik GmbH).

Subjects were instructed not to exercise or drink alcohol 48 h before the intervention period. On the 4 days before the respective intervention days, subjects completed 1-d dietary and activity records. Dietary records were analyzed using the nutrient calculation program EBISpro (University of Hohenheim, Stuttgart, Germany) based on the German Nutrient Database Bundeslebensmittelschlüssel (Max-Rubner Institute, Karlsruhe, Germany). For activity records, the metabolic equivalent of tasks was calculated [18]. In addition, subjects were asked to record the time they went to bed and woke up during the study days, from which the duration of sleep was calculated. During the measurements, subjects were awake and lay quietly and motionless. Females were tested in the luteal phase of their menstrual cycle or took ovulation-suppressing oral contraceptives (*n* = 4) to avoid an effect of the menstrual cycle on REE.

### Measurements

Subjects were asked to appear fasted (10 h) and rested on the mornings of examinations. Additionally, they were instructed to wear the same short sportswear on each study day. Before they entered the intervention room, subjects were exposed to an ambient temperature of 20–22 °C for 15 min to measure their body weight (Jura Personenwaage; Soehnle) and BP (Boso Carat Professional; Bosch + Sohn GmbH).

After entering the intervention room, subjects lied down on a medical couch for a 30-min resting period to acclimatize to the ambient temperature before the measurement onset. During this period, sensors for measuring body core and body surface temperatures were installed. Body core temperature was measured with a contact technique in the ear canal (Three; Cosinuss GmbH). Body surface temperature was measured with surface sensors, which were attached to the skin surface (ALMEMO 809 V7; Ahlborn Mess- und Regelungstechnik). Both measurements were carried out according to ISO 9886-2004 [19]. Values were collected every 10 s based on the measurement interval of gas analysis. Furthermore, bioelectric impedance analysis (Data Input GmbH) was performed on each study day to determine body composition.

After the acclimatization period, REE and substrate oxidation were determined by gas analysis for 20 min. VO_2_ and VCO_2_ were determined every 10 s using a ventilated canopy system (Quark-RMR; COSMED). A mass flow sensor measured volume and airflow. Flow and gas analyzers were calibrated before each intervention measurement. VO_2_ and VCO_2_ were converted to REE using the abbreviated Weir equation [20]. Substrate oxidation was determined by the respiratory quotient (RQ) of VCO_2_ and VO_2_. Together with REE and RQ, HR was measured using a chest belt (Herzfrequenz Messsystem; SmartLAB), which was wirelessly connected to the Quark-RMR device. Body core temperature, HR and body surface temperature were measured simultaneous with indirect calorimetry.

Moreover, subjects assessed their subjective temperature perception using the visual analog scale when entering and leaving the intervention room. The visual analog scale to determine subjective temperature perception used a 10-point Likert scale [21,22]. After all examinations, subjects left the study center until the next study day.

### Statistical analysis

To our knowledge, as this is the first randomized crossover study conducted using indirect calorimetry via canopy to determine the influence of different ambient temperatures on REE, no precise data were available on which a sample size calculation could have been based upon. Effect size was estimated on the basis of the available studies by Westerterp-Plantega et al. [12,13]. With a 1-sided significance level of 0.05 and an intrapersonal SD of 0.5, 0.7, and 0.9 MJ/d, the detectable differences with an 80% power are also 0.5, 0.7, and 0.9 MJ/d. For this reason, the number of cases was planned with 28 evaluable cases in the 4 × 4 Williams design and 32 subjects (assuming a dropout rate of 10%) were included in the study.

During indirect calorimetry, measurements of VO_2_ and VCO_2_ were taken every 10 s. During data processing of indirect calorimetry, data from the first 5 min and some outliers obtained during indirect calorimetry data collection were removed. An outlier was defined as a value that deviated >10% from the mean value. Outliers were removed using the dual control principle. Subsequently, mean VO_2_ and VCO_2_ values were used to calculate mean REE and RQ for statistical analyses. In figures, data points were defined as outliers if they deviated from the quartile by >1.5 times the IQR. However, these values were not excluded from statistical analysis, as they could be explained physiologically.

All statistical analyses were performed using SPSS version 29. Initially, parameters that significantly influenced the respective outcome were identified in a multiple regression model with forward variable selection. Following this, changes in REE, RQ, VO_2_, VCO_2_, HR, body core, and surface temperatures and subjective temperature perception among the 4 interventions were tested for significance in univariate linear mixed models. The visit was selected as the repeating factor, the covariance type for repeated measures was composite symmetrical heterogeneous, and ambient temperature and sex were set as design parameters in each model. Covariates were previously defined in a forward multiple linear regression including all measured variables. For REE, VO_2_ and VCO_2_, FFM, and HR were identified as relevant covariates. For HR, age, height, body core temperature, and diastolic BP were identified as relevant covariates. Except for ambient temperature, no factor significantly influenced RQ. To test mediation between REE and HR, body core temperature, and ambient temperature, mediation analysis was performed according to Hayes and Preacher [23]. As there was an indirect effect of ambient temperature on heart rate (c = 0.5204; *P* < 0.001), HR was specified as an outcome in a separate linear mixed model and excluded from the main model. Significant differences of baseline characteristics between sexes were analyzed using the paired *t* test. Differences in diet, activity and sleep between the days before the intervention and differences in body weight, body composition and resting BP between each intervention morning were analyzed using a single-factor ANOVA. Pearson correlation analyses were carried out for all metric variables. The level of significance was set to *P* < 0.05 (two-tailed). Results are presented as mean ± standard error of mean if not otherwise indicated.

## Results

Activity, energy and nutrient intake, and sleep duration did not significantly differ between the days before the intervention. In addition, body weight, body composition, and resting BP did not significantly differ between the 4 intervention days (Supplemental Table 1).

### Influence of ambient temperature on respiratory analysis

FFM, HR, and ambient temperature (all *P* < 0.001) were the most important factors influencing REE in the forward linear regression model. They explained 61.3% of the variance in REE and were also the most important factors influencing VCO_2_ (FFM and ambient temperature: *P* < 0.001; HR: *P* = 0.002) and VO_2_ (FFM and HR: *P* < 0.001; ambient temperature: *P* = 0.004).

**FIGURE 2:**
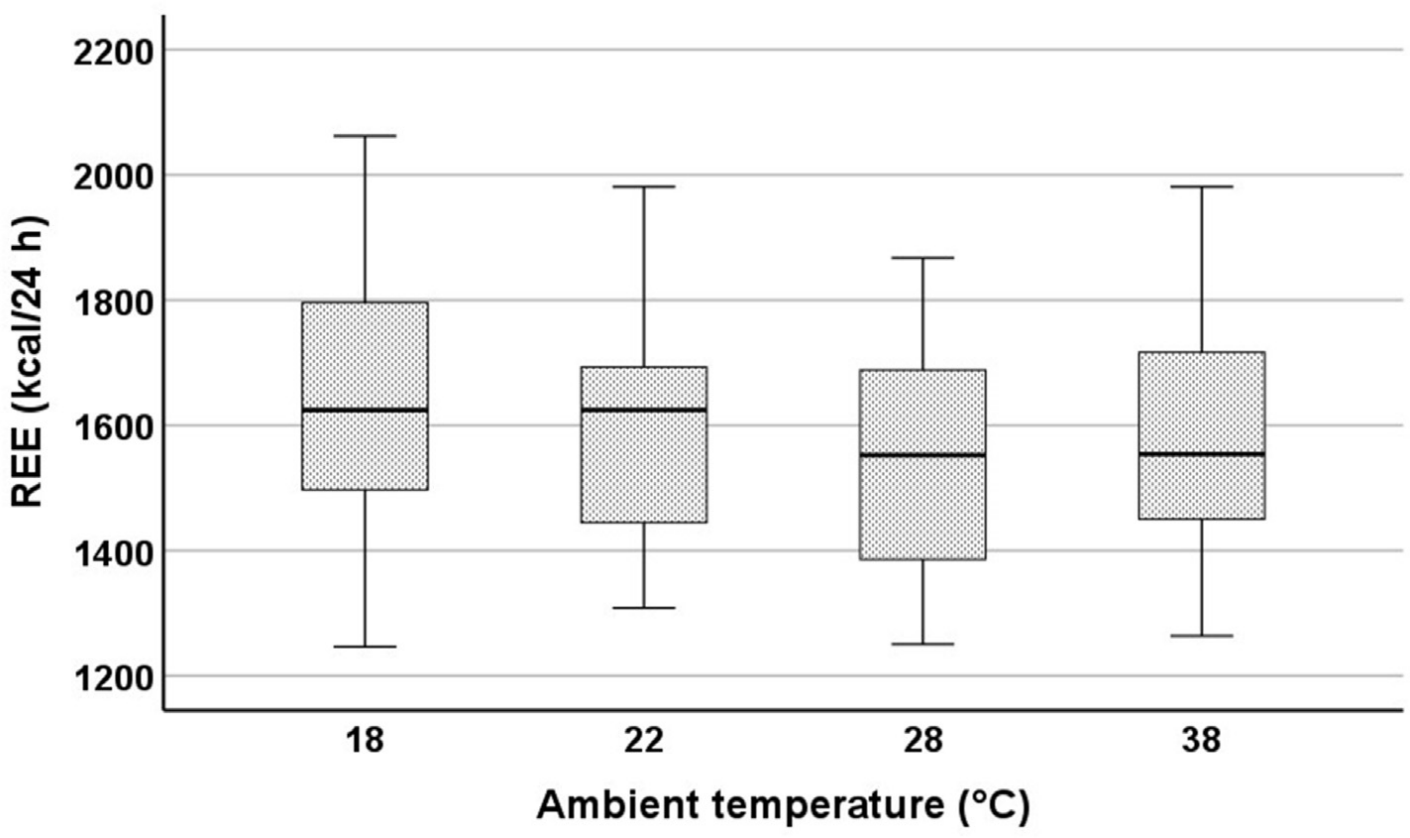
REE (kcal/24 h) of the subjects at 18, 22, 28, and 38 °C. REE, resting energy expenditure.

**FIGURE 3:**
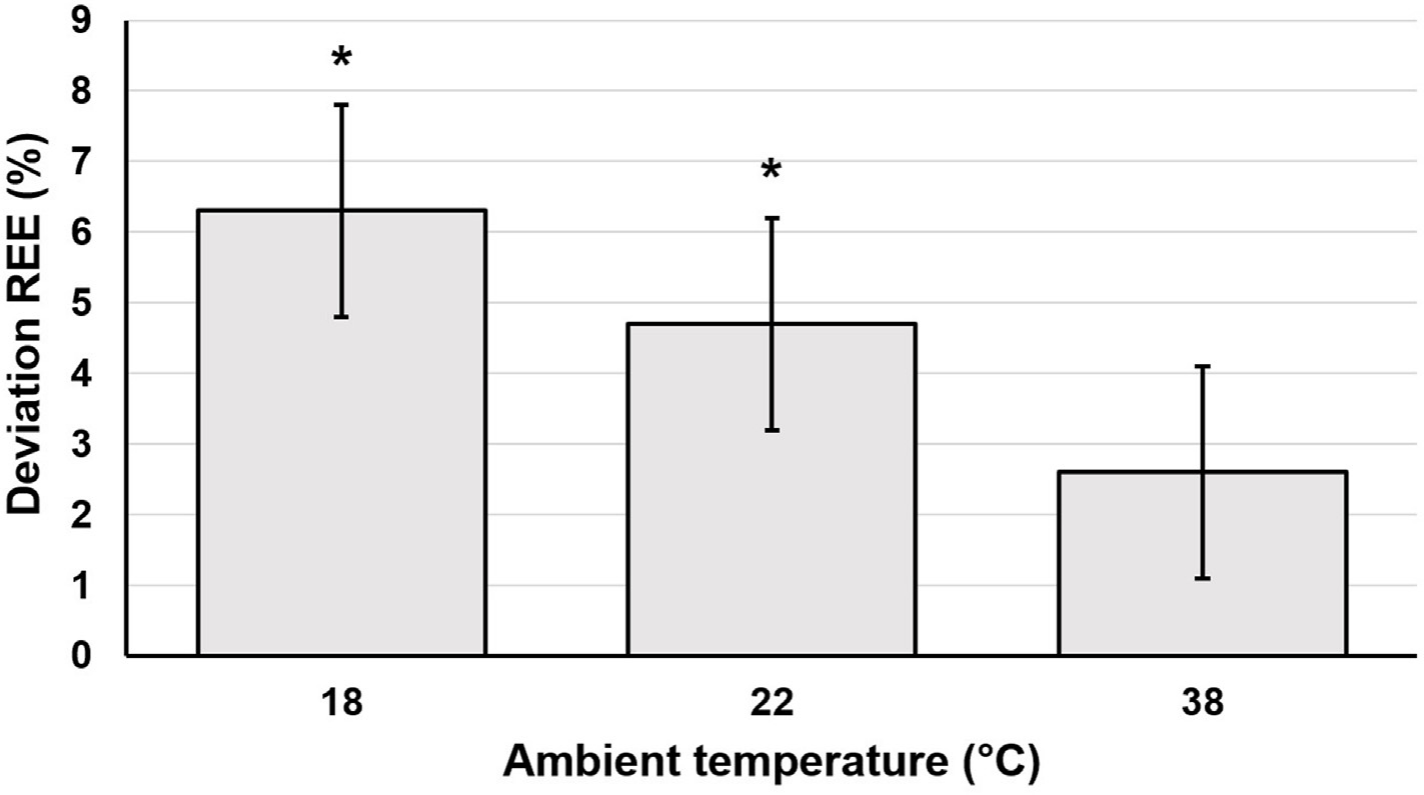
Percentage deviation of REE at different ambient temperatures compared with that at 28 °C. (Values are presented as mean ± SEM). ∗The difference is significant at a significance level of α = 0.05. REE, resting energy expenditure.

**FIGURE 4:**
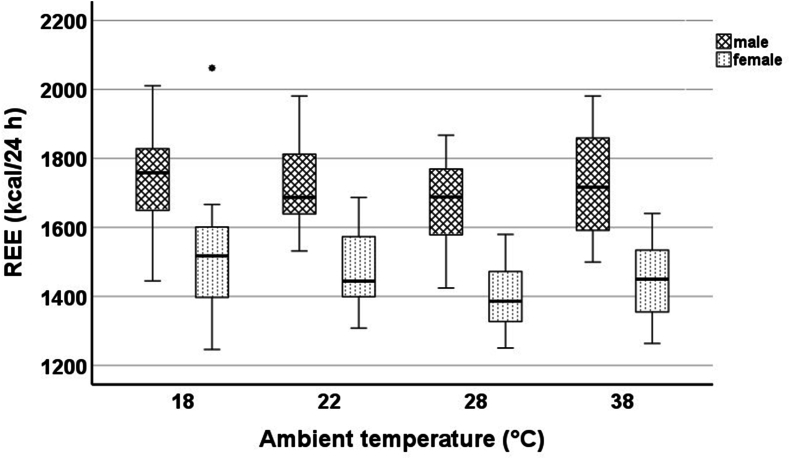
REE (kcal/24 h) of the subjects at 18, 22, 28, and 38 °C subdivided according to sexes. ∗Outliers. REE, resting energy expenditure.

In the linear mixed model, REE significantly differed between 18 and 28 °C (18 °C: +96 ± 24 kcal/24 h; *P* < 0.001), 18 and 38 °C (18 °C: +57 ± 23 kcal/24 h; *P* = 0.016), and 22 and 28 °C (22 °C: +73 ± 24 kcal/24 h; *P* = 0.003). Figure 2 shows the absolute REE values at different ambient temperatures represented as boxplots (Supplemental Figure 1 and Supplemental Tables 2 and 3). Table 2 shows the absolute differences and Figure 3 the percentage differences in REE between the different ambient temperatures. Differences in REE at different ambient temperatures were similar for both sexes (Figure 4). Taking FFM into account, sex had, in contrast to ambient temperature, no significant influence on REE. Based on the ambient temperature (thermoneutral) of 28 °C, the following regression formula was created for REE:

REE(kcal/24h)(28°C)=781+15.87×LBM(kg)−61(females)+96(18°C)+73(22°C)+39(38°C)

**TABLE 2:** Absolute differences in REE (kcal/24 h) between different intervention temperatures.

Ambient temperature (°C)	REE difference (kcal/24 h)		*P*	95% CI (kcal/24 h)	
	Mean	SEM		Lower limit	Upper limit
18 compared with 22	23	23	0.319	−23	70
18 compared with 28	96	23	<0.001^1^	49	143
18 compared with 38	57	23	0.016^2^	11	104
22 compared with 28	73	23	0.003^3^	26	119
22 compared with 38	34	23	0.152	−13	81
28 compared with 38	−39	23	0.101	−85	8

*Abbreviations:* REE, resting energy expenditure.

1 *P* < 0.001 (linear mixed model).

2 *P* < 0.05 (linear mixed model).

3 *P* < 0.01 (linear mixed model).

VO_2_ significantly differed between 22 and 28 °C (22 °C: +21 ± 7 mL/min; *P* = 0.005) and VCO_2_ between 18 and 38 °C (18 °C: +13 ± 6 mL/min; *P* = 0.024) (Supplemental Figures 2 and 3). There was a correlation between ambient temperature and VCO_2_ (*r* = −0.215; *P* = 0.015).

RQ was lower at 38 °C than that at all other ambient temperatures (38 compared with 18 °C: −0.4 ± 0.009; *P* < 0.001; 38 compared with 22 °C: −0.28 ± 0.009; *P* = 0.003; 38 compared with 28 °C: −0.3 ± 0.009; *P* = 0.002) (data not shown).

### Influence of ambient temperature on HR

**FIGURE 5:**
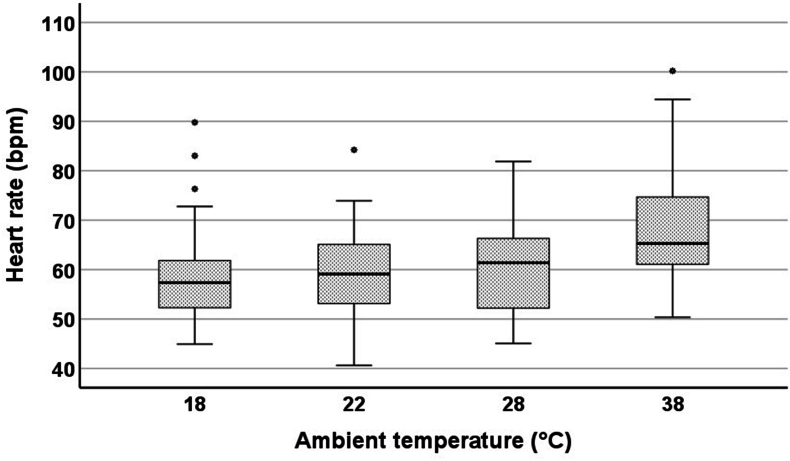
Boxplots of heart rate (absolute values) at different ambient temperatures. ∗Outliers.

There was a linear correlation between ambient temperature and HR (*r* = 0.348; *P* < 0.001) and between body core temperature and HR (*r* = 0.426; *P* < 0.001). The relationship between ambient temperature and HR was mediated by body core temperature. HR significantly differed between 28 and 18 °C (2.0 ± 1.0 bpm; *P* = 0.04) and between 38 °C and all other ambient temperatures (38 compared with 28 °C: 8.0 ± 1.0 bpm; 38 compared with 22 °C: 9.6 ± 1.0 bpm; 38 compared with 18 °C: 10.0 ± 1.0 bpm; all *P* < 0.001) (Supplemental Tables 2 and 3 and Figure 5).

### Correlations between different physiologic characteristics at different ambient temperatures

**FIGURE 6:**
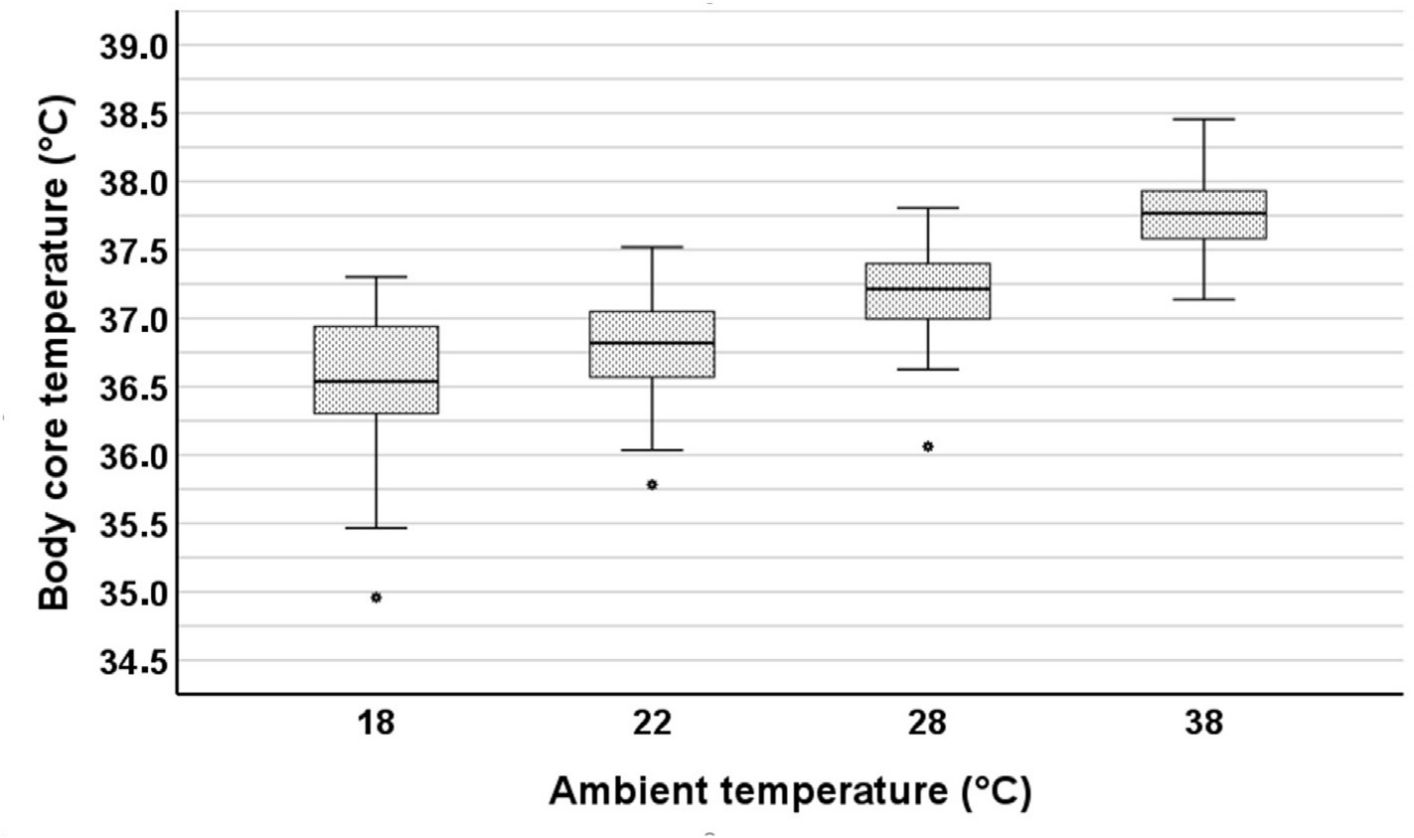
Boxplots of body core temperature at different ambient temperatures. ∗Outliers.

There were strong linear correlations between ambient temperature and body core temperature (*r* = 0.765; *P* < 0.001) (Figure 6), body surface temperature (*r* = 0.937; *P* < 0.001), and subjective temperature perception after the intervention (*r* = 0.882; *P* < 0.001). All these parameters differed across all ambient temperatures (all *P* < 0.001) (Supplemental Table 4).

FM (*r* = −0.187; *P* = 0.036) and HR (*r* = 0.298; *P* < 0.001) correlated with subjective temperature perception after the intervention. FM also correlated with the difference between body core and body surface temperatures (*r* = 0.232; *P* = 0.008).

## Discussion

Previous studies indicated that REE changes with long-term exposure to different temperatures. The recommended ambient temperature for indirect calorimetry via canopy is 20–25 °C with light clothing [17]. However, there is no scientific evidence on the effects of short-term temperature intervention on REE. Therefore, the aim of this randomized crossover study was to investigate the short-term influence of different ambient temperatures on REE measured by indirect calorimetry via canopy method. The hypothesis that REE was the lowest at a temperature corresponding to the thermoneutral zone of humans (28 °C) was partially confirmed. REE was significantly higher at 18 and 22 °C than that at 28 °C but did not differ significantly between 28 and 38 °C.

### Influence of cold temperatures on REE

Previous literature evaluating the influence of various ambient temperatures on TEE over an extended period suggests that TEE is significantly lower at an ambient temperature, corresponding to the thermoneutral zone than at a temperature ≥5 °C cooler [3,4,[8], [9], [10], [11], [12], [13]]. However, this study, which performed indirect calorimetry with a canopy to examine REE, differs from previous studies conducted in respiration chambers over a long period, where strict activity protocols were imposed on participants. A previous study reported that REE was higher at 18 °C than that at 24 °C upon 1 exposure for 2.5 h [7].

REE differed by 96 kcal/24 h between 18 and 28 °C in this present study. Higher muscle tone and shivering in response to cold significantly increase REE due to the increased adenosine triphosphate production required for intensified muscle contractions [24]. Compared with other organs, the proportion of muscles in FFM is relatively large [2,25]. Therefore, increased muscle tone increases REE, which is already detectable after a short period, as the present model suggests. In 1 female participant in our study, REE was 39% higher at 18 °C than that at 38 °C, which was attributed to shivering during the entire gas analysis.

Another explanation for an increase in REE can be an activation of the sympathetic nervous system. There is a close relationship among an increased triiodothyronine concentration, sympathetic nervous system, and energy expenditure [26]. Activation of sympathetic nervous system can already occur in short term as a result of thermal stress [27], but no blood biomarkers were collected in this study to confirm this assumption.

Cold-induced conversion of white adipose tissue (WAT) to brown adipose tissue (BAT) or activation of BAT can also contribute to an increase of energy expenditure. BAT has significantly more mitochondria than WAT, and uncoupling protein-1 in the mitochondrial membrane induces proton leakage, releasing energy in the form of heat. Short-term cold exposure activates BAT, whereas long-term cold exposure increases conversion of WAT to BAT [28]. BAT may increase REE by ≤20% during cold exposure, sustaining research interest in cold-induced thermogenesis and its long-term effects on weight loss [29]. However, as BAT activity was not measured in this study, the specific contributions of these processes to the increase of REE cannot be precisely determined.

### Influence of heat on REE and HR

The Q_10_-effect (or temperature coefficient) describes the ratio of a physiologic parameter at a certain temperature to the same parameter at a temperature of 10 K cooler [30]. Biochemical processes are accelerated at higher temperatures due to increased kinetic energy, which speeds up physiologic processes [31,32]. Although it was hypothesized that REE might increase at a certain temperature, this study did not observe an increase of REE at 38 °C compared with that at 28 °C.

However, there was a positive linear correlation between ambient temperature and HR. After a short exposure time, HR was significantly higher at 38 °C than at all other ambient temperatures. This aligns with existing research and can be explained by the Q_10_-effect [33,34]. Vascular dilation increases heat dissipation to the surface, and blood circulates more rapidly from the warmer core to the outer periphery with an elevated HR, accelerating heat dissipation [34,35], which can also be a result of activation of the sympathetic nervous system [27].

Despite the positive correlation between ambient temperature and HR, REE was not significantly higher at 38 °C than that at 28 °C. As the heart alone contributes ∼11.9% to REE [25,36], we assume that increased energy expenditure due to an increased HR was too small to detect in our study. When energy is converted in muscle, 40% of total energy is released into the system in the form of heat [29]. Vigorous exercise at high temperatures further increases the intramuscular temperature in proportion to the workload, which is why exercise under the influence of heat can cumulatively increase TEE [37]. In the present model, short-term exposure to heat did not show any effects on REE.

In a review of the literature regarding the impact of heat on REE, Henderson and Halsey [38] found heterogeneous results from studies that expose subjects to passive hyperthermia. Studies in which ambient temperature was continuously increased did not observe a significant increase of REE at high temperatures, possibly due to a carryover effect. Some studies that maintain an unaltered hot ambient temperature observed an increase of REE. In general, the study designs were heterogeneous and had small sample sizes (*n* ≤ 15). The authors also noted a lack of information about the general health and body composition of the individuals [38]. Therefore, further research regarding the influence of heat on REE is necessary.

In 2021, Henderson et al. [16] conducted an intervention study to define an upper limit above the thermoneutral zone where REE begins to rise. They also sought to understand the underlying physiologic mechanisms. VO_2_ significantly differed between baseline and postintervention. Compared with baseline and all other conditions, VO_2_ and HR increased substantially at 50 °C and 50% relative humidity. Above this temperature, humidity seemed to have a cumulative effect on heat stress and heat dissipation mechanisms. However, this study had methodologic limitations, such as including a heterogeneous subject group and excluding 6 of the 13 subjects due to measurement errors during indirect calorimetry. In addition, there is a lack of information about how exactly the metabolic rate was calculated [16].

Although the sample was homogeneous, there was biological variability in the anthropometric data and REE in this study (Figure 2). Additionally, an individual’s thermoregulation depends on various factors, such as body composition [39]. In our study, there was a positive linear correlation between FM and the difference between core and surface body temperatures. A higher percentage of FM slows heat dissipation through convection, potentially leading to a lower thermoneutral zone in individuals with higher FM [40]. This may explain why REE of some subjects with higher FM was the lowest at temperatures below 28 °C in our study.

### Strengths and limitations

This study highlighted the advantages of indirect calorimetry using a canopy and underscored the importance of conducting measurements at controlled ambient temperatures for (clothed) subject even for exposure to different ambient temperatures for short periods. The randomized crossover design and control of ambient (temperature and humidity) and physiologic (BP, nutrition, activity, sleep, menstrual cycle, and especially weight and body composition) conditions minimized confounding factors. The measurements were conducted over a short 4-d period, minimizing participant burden and achieving 100% compliance, indicate that the data are of high quality. The topic of this study is of highly practical relevance, and to our knowledge, this study is one of the few to investigate the influence of heat on REE.

Indirect calorimetry using a canopy has limitations, requiring calm and evenly breathing subjects. At 38 °C, breathing was noticeably more erratic due to the uncomfortable heat under the canopy, leading to outliers that were not considered in the final analysis. Nevertheless, owing to the high practicability, we deliberately chose indirect calorimetry using a canopy in this study. The aim of this study was to test short-term influence of ambient temperature on REE in indirect calorimetry using a canopy. To better understand the physiologic impact of prolonged heat on REE, future studies conducted in respiration chambers or using the doubly labeled water method might be more appropriate to reveal more pronounced effects of heat exposure on energy expenditure by increasing the ambient temperature or extending the intervention duration.

### Conclusion

In this study, mild cold intervention at 18 and 22 °C for a short period significantly increased REE compared with a thermoneutral ambient temperature of 28 °C. Heat exposure at 38 °C did not increase or decrease REE compared with 28 °C. The immediate influence of cold on REE underscores the importance of conducting indirect calorimetry using a canopy under controlled ambient conditions.

## Author contributions

The authors’ responsibilities were as follows—SE, DP: provided funding; SH, PF-M, CD, DP, SE: designed the study; SH, CD, BS-W, MC, SE: recruited the subjects and performed the study; SH, PF-M, BS-W: participated in data collection and analysis; SH, CD, RN: performed the statistical calculations; SH, CD, SE: wrote the manuscript; DP, PF-M, BS-W, RN: contributed to the final discussion of the results; and all authors: have read and approved the final manuscript.

## Data availability

Data will be made available upon reasonable request.

## Funding

This research received no specific grant from any funding agency, commercial or not-for-profit sectors.

## Conflict of interest

The authors report no conflicts of interest.
