# Supplementary material for: Influence of Ambient Temperature on Resting Energy Expenditure in Metabolically Healthy Males and Females
Source: J Nutr. 2025 Jan 13;155(3):862–70. doi: 10.1016/j.tjnut.2025.01.013 (PMC11934286; doi:10.1016/j.tjnut.2025.01.013)
Supplement: Multimedia component 1 [file mmc1.docx]

**Influence of ambient temperature on resting energy expenditure in metabolically healthy men and women**

**Henkel, Sara**

**Egert, Sarah (corresponding author)**

**Supplementary Table 1:** Control parameters on different study days

|  | Day1 (n=32) | | | Day 2 (n=32) | | | Day 3 (n=32) | | | Day 4 (n=32) | |
| --- | --- | --- | --- | --- | --- | --- | --- | --- | --- | --- | --- |
|  | Mean | SD | Mean | | SD | Mean | | SD | Mean | | SD |
| Body weight (kg) | 68.6 | 8.0 | 68.7 | | 8.1 | 68.6 | | 8.0 | 68.4 | | 8.1 |
| FFM (kg) | 51.3 | 7.9 | 51.5 | | 7.7 | 51.5 | | 7.9 | 51.8 | | 7.9 |
| FM (kg) | 17.2 | 4.8 | 17.3 | | 4.7 | 17.1 | | 5.1 | 16.7 | | 4.9 |
| FM (%) | 25.2 | 6.8 | 25.1 | | 6.4 | 24,9 | | 7.2 | 24.4 | | 6.9 |
| Resting systolic blood pressure (mmHg) | 125 | 12 | 122 | | 11 | 121 | | 11 | 119 | | 10 |
| Resting diastolic blood pressure (mmHg) | 81 | 7 | 77 | | 9 | 78 | | 7 | 77 | | 6 |
| Resting pulse rate (bpm) | 70 | 11 | 68 | | 12 | 68 | | 12 | 67 | | 11 |
| Activity (METs) | 1.5 | 0.1 | 1.6 | | 0.2 | 1.6 | | 0.2 | 1.6 | | 0.1 |
| Energy intake (kcal/d) | 2324 | 835 | 2089 | | 635 | 2266 | | 744 | 2248 | | 610 |
| Fat intake (g/d) | 107 | 54 | 90 | | 44 | 104 | | 49 | 99 | | 39 |
| Carbohydrate intake (g/d) | 236 | 80 | 224 | | 65 | 240 | | 93 | 238 | | 68 |
| Protein intake (g/d) | 99 | 46 | 81 | | 32 | 85 | | 29 | 90 | | 38 |
| Sleep duration (h) | 7.0 | 1.0 | 7.0 | | 0.8 | 7.0 | | 1.0 | 7.0 | | 0.9 |

**Supplementary Table 2:** Estimates of fixed parameters in linear mixed models

| **Parameter** | **coefficient** | **SEM** | | | **df** | | **T** | | **p-value** | | **95 % confidence interval** | | | |
| --- | --- | --- | --- | --- | --- | --- | --- | --- | --- | --- | --- | --- | --- | --- |
|  |  |  |  |  |  |  |  |  |  |  | **lower** | | **upper** | |
| **Model: Resting energy expenditure** | | | | | | | | | | | | | | |
| Constant term | 780.76 | 215.18 | | | 34.179 | | 3.628 | | <0.001 | | 343.56 | | 1217.97 | |
| Fat free mass (kg) | 15.87 | 3.66 | | | 34.086 | | 4.341 | | <0.001 | | 8.44 | | 23.30 | |
| Sex female | -61.38 | 57.05 | | | 32.649 | | -1.076 | | 0.290 | | -177.50 | | 54.74 | |
| Sex male | 0 | 0 | | | - | | - | | - | | - | | - | |
| Ambient temperature 18°C | 57.30 | 24.37 | | | 89.235 | | 2.352 | | 0.021 | | 8.89 | | 105.72 | |
| Ambient temperature 22°C | 33.87 | 24.43 | | | 88.162 | | 1.387 | | 0.169 | | -14.68 | | 82.42 | |
| Ambient temperature 28°C | -38.70 | 24.31 | | | 88.231 | | -1.592 | | 0.115 | | -87.02 | | 9.61 | |
| Ambient temperature 38°C | 0 | 0 | | | - | | - | | - | | - | | - | |
| **Model: Heart rate** |  |  | | |  | |  | |  | |  | |  | |
| Constant term | 135.24 | 53.17 | | | 25.679 | | 2.544 | | 0.017 | | 25.89 | | 244.59 | |
| Age (years) | 0.79 | 0.63 | | | 25.702 | | 1.240 | | 0.226 | | -0.52 | | 2.10 | |
| Height (cm) | -0.56 | 0.32 | | | 26.560 | | -1.773 | | 0.088 | | -1.21 | | 0.09 | |
| Diastolic blood pressure (bpm) | 0.16 | 0.09 | | | 102.181 | | 1.774 | | 0.079 | | -0.02 | | 0.35 | |
| Sex female | -1.57 | 3.92 | | | 25.669 | | -0.401 | | 0.692 | | -9.64 | | 6.50 | |
| Sex male | 0 | 0 | | | - | | - | | - | | - | | - | |
| Ambient temperature 18°C | -10.02 | 1.00 | | | 89.848 | | -10.07 | | <0.001 | | -11.99 | | -8.04 | |
| Ambient temperature 22°C | -9.62 | 1.01 | | | 90.045 | | -9.573 | | <0.001 | | -11.62 | | -7.62 | |
| Ambient temperature 28°C | -8.04 | 1.02 | | | 90.977 | | -7.910 | | <0.001 | | -10.06 | | -6.02 | |
| Ambient temperature 38°C | 0 | 0 | | | - | | - | | - | | - | | - | |
| **Model: Body core temperature** | | |  |  | |  | |  | |  | |  | |  |
| Constant term | 35.10 | 1,57 | | | 34.200 | | 22.332 | | <0.001 | | 31.91 | | 38.30 | |
| Height (cm) | 0.01 | 0.01 | | | 32.752 | | 1,521 | | 0.138 | | -0.01 | | 0.03 | |
| Sleep duration (h) | 0.08 | 0.04 | | | 100.703 | | 1.958 | | 0.053 | | 0 | | 0.16 | |
| Ambient humidity (%) | -0.01 | 0.01 | | | 93.459 | | -1.796 | | 0.076 | | -0.02 | | 0 | |
| Sex female | 0.13 | 0.11 | | | 33.103 | | 1.138 | | 0.263 | | -0.1 | | 0.36 | |
| Sex male | 0 | 0 | | | - | | - | | - | | - | | - | |
| Ambient temperature 18°C | -1.09 | 0.09 | | | 91.260 | | -11.624 | | <0.001 | | -1.28 | | -0.91 | |
| Ambient temperature 22°C | -0.84 | 0.09 | | | 101.414 | | -9.014 | | <0.001 | | -1.02 | | -0.65 | |
| Ambient temperature 28°C | -0.58 | 0.07 | | | 90.037 | | -7.790 | | <0.001 | | -0.72 | | -0.43 | |
| Ambient temperature 38°C | 0 | 0 | | | - | | - | | - | | - | | - | |
| **Model: Body surface temperature** | | | | | | | | | | | | | | |
| Constant term | 36.37 | 0.74 | | | 28.932 | | 49.246 | | <0.001 | | 34.86 | | 37.88 | |
| Fat mass (kg) | -0.04 | 0.02 | | | 34.441 | | -2.215 | | 0.033 | | -0.08 | | 0 | |
| Age (years) | 0.03 | 0.03 | | | 28.924 | | 1.124 | | 0.270 | | -0.03 | | 0.09 | |
| Sex female | -0.05 | 0.20 | | | 30.612 | | -0.264 | | 0.794 | | -0.46 | | 0.36 | |
| Sex male | 0 | 0 | | | - | | - | | - | | - | | - | |
| Ambient temperature 18°C | -4.41 | 0.10 | | | 85.490 | | -42.374 | | <0.001 | | -4.61 | | -4.20 | |
| Ambient temperature 22°C | -3.30 | 0.10 | | | 84.815 | | -31.917 | | <0.001 | | -3.50 | | -3.09 | |
| Ambient temperature 28°C | -1.78 | 0.10 | | | 85.840 | | -17.241 | | <0.001 | | -1.99 | | -1.58 | |
| Ambient temperature 38°C | 0 | 0 | | | - | | - | | - | | - | | - | |
| **Model: Respiratory Quotient** | | | | | | | | | | | | | | |
| Constant term | 0.76 | 0.01 | | | 67.216 | | 76.533 | | <0.001 | | 0.74 | | 0.78 | |
| Sex female | -0.01 | 0.02 | | | 92.068 | | -0.678 | | 0.499 | | -0.04 | | 0.02 | |
| Sex male | 0 | 0 | | | - | | - | | - | | - | | - | |
| Ambient temperature 18°C | 0.03 | 0.01 | | | 78.198 | | 2.037 | | 0.045 | | 0 | | 0.05 | |
| Ambient temperature 22°C | 0.03 | 0.01 | | | 79.854 | | 1.929 | | 0.057 | | 0 | | 0.05 | |
| Ambient temperature 28°C | 0.03 | 0.01 | | | 79.992 | | 1.872 | | 0.065 | | 0 | | 0.05 | |
| Ambient temperature 38°C | 0 | 0 | | | - | | - | | - | | - | | - | |

SEM, standard error of mean, df, degrees of freedom; T, ratio of coefficient to SEM

**Supplementary Table 3:** Comparison of parameters between interventions

| **Ambient temperature** | **mean difference** | **SEM** | **df** | **p-value** | **95 % confidence interval** | |
| --- | --- | --- | --- | --- | --- | --- |
|  |  |  |  |  | **lower** | **upper** |
| **Model: Resting energy expenditure** | | | | | | |
| 18°C vs. 22°C | 23.43 | 23.36 | 88.383 | 0.319 | -22.99 | 69.85 |
| 18°C vs. 28°C | 96.01 | 23.47 | 87.742 | <0.001 | 49.36 | 142.65 |
| 18°C vs. 38°C | 57.30 | 23.40 | 89.235 | 0.016 | 10.81 | 103.79 |
| 22°C vs. 28°C | 72.58 | 23.47 | 89.372 | 0.003 | 25.95 | 119.20 |
| 22°C vs. 38°C | 33.87 | 23.45 | 88.162 | 0.152 | -12.73 | 80.47 |
| 28°C vs. 38°C | -38.70 | 23.35 | 88.231 | 0.101 | -85.10 | 7.69 |
| **Model: Heart rate** | | | | | | |
| 18°C vs. 22°C | -0.40 | 0.95 | 89.941 | 0.678 | -2.28 | 1.49 |
| 18°C vs. 28°C | -1.98 | 0.95 | 89.400 | 0.040 | -3.86 | -0.09 |
| 18°C vs. 38°C | -10.02 | 0.95 | 89.85 | <0.001 | -11.91 | -8.12 |
| 22°C vs. 28°C | -1.58 | 0.95 | 90.312 | 0.099 | -3.47 | 0.31 |
| 22°C vs. 38°C | -9.62 | 0.96 | 90.045 | <0.001 | -11.53 | -7.71 |
| 28°C vs. 38°C | -8.04 | 0.97 | 90.977 | <0.001 | -9.97 | -6.11 |
| **Model: Body core temperature** | | | | | | |
| 18°C vs. 22°C | -0.25 | 0.07 | 82.511 | <0.001 | -0.38 | -0.13 |
| 18°C vs. 28°C | -0.52 | 0.07 | 86.931 | <0.001 | -0.66 | -0.37 |
| 18°C vs. 38°C | -1.09 | 0.09 | 91.260 | <0.001 | -1.27 | -0.91 |
| 22°C vs. 28°C | -0.26 | 0.07 | 91.793 | <0.001 | -0.40 | -0.12 |
| 22°C vs. 38°C | -0.84 | 0.09 | 101.414 | <0.001 | -1.01 | -0.66 |
| 28°C vs. 38°C | -0.58 | 0.07 | 90.037 | <0.001 | -0.72 | -0.44 |
| **Model: Body surface temperature** | | | | | | |
| 18°C vs. 22°C | -1.11 | 0.10 | 85.291 | <0.001 | -1.31 | -0.91 |
| 18°C vs. 28°C | -2.62 | 0.10 | 85.007 | <0.001 | -2.82 | -2.43 |
| 18°C vs. 38°C | -4.41 | 0.10 | 85.490 | <0.001 | -4.61 | -4.21 |
| 22°C vs. 28°C | -1.52 | 0.10 | 83.009 | <0.001 | -1.71 | -1.32 |
| 22°C vs. 38°C | -3.30 | 0.10 | 84.815 | <0.001 | -3.50 | -3.10 |
| 28°C vs. 38°C | -1.78 | 0.10 | 85.840 | <0.001 | -1.98 | -1.59 |
| **Model: Respiratory Quotient** | | | | | | |
| 18°C vs. 22°C | 0.01 | 0.01 | 85.870 | 0.200 | -0.01 | 0.03 |
| 18°C vs. 28°C | 0.01 | 0.01 | 85.985 | 0.275 | -0.01 | 0.03 |
| 18°C vs. 38°C | 0.04 | 0.01 | 84.557 | <0.001 | 0.02 | 0.06 |
| 22°C vs. 28°C | 0 | 0.01 | 87.126 | 0.855 | -0.02 | 0.02 |
| 22°C vs. 38°C | 0.03 | 0.01 | 82.386 | 0.003 | 0.01 | 0.05 |
| 28°C vs. 38°C | 0.03 | 0.01 | 86.311 | 0.002 | 0.01 | 0.05 |

SEM, standard error of mean; df, degrees of freedom

**Supplementary Table 4:** Test parameters measured at different intervention temperatures

|  | 18°C (n=32) | | | 22°C (n=32) | | | 28°C (n=32) | | | 38°C (n=32) | |
| --- | --- | --- | --- | --- | --- | --- | --- | --- | --- | --- | --- |
|  | Mean | SEM | Mean | | SEM | Mean | | SEM | Mean | | SEM |
| REE (kcal/24 h)* | 1625 | 21 | 1602 | | 21 | 1529 | | 21 | 1568 | | 21 |
| RQ** | 0.79 | 0.01 | 0.78 | | 0.01 | 0.78 | | 0.01 | 0.75 | | 0.01 |
| Heart rate (bpm)*** | 59 | 2 | 60 | | 2 | 61 | | 2 | 69 | | 2 |
| VO_2_ (mL/min)† | 227 | 7 | 239 | | 7 | 219 | | 6 | 228 | | 6 |
| VCO_2_ (mL/min)†† | 187 | 6 | 185 | | 5 | 177 | | 5 | 174 | | 5 |
| Body core temperature (°C)††† | 36.6 | 0.1 | 36.9 | | 0.1 | 37.1 | | 0.1 | 37.7 | | 0.1 |
| Body surface temperature (°C)††† | 32.0 | 0.1 | 33.1 | | 0.1 | 34.6 | | 0.1 | 36.4 | | 0.1 |

* 18°C vs. 28°C: P<0.001; 18°C vs. 38°C: P=0.016; 22°C vs. 28°C: P=0.003
** 18°C vs. 38°C: P<0.001; 22°C vs. 38°C: P=0.003; 28°C vs. 38°C: P=0.002
***18°C vs. 28°C: P=0.04; 18°C, 22°C, 28°C vs. 38°C: all P<0.001
† 22°C vs. 28°C: P=0.005
†† 18°C vs. 38°C: P=0.024
††† all P<0.001
