# Supplementary material for: Influence of Ambient Temperature on Resting Energy Expenditure in Metabolically Healthy Males and Females
Source: J Nutr. 2025 Jan 13;155(3):862–70. doi: 10.1016/j.tjnut.2025.01.013 (PMC11934286; doi:10.1016/j.tjnut.2025.01.013)
Supplement: Multimedia component 2 [file mmc2.docx]

**Influence of ambient temperature on resting energy expenditure in metabolically healthy men and women**

**Henkel, Sara**

**Egert, Sarah (corresponding author)**

**Supplementary Figure 1**


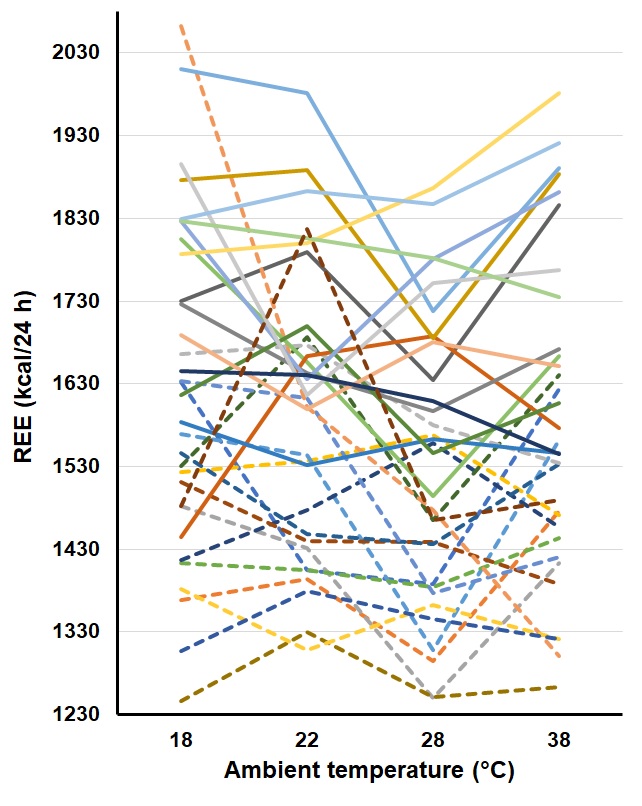


**Supplementary Figure 1:** Individual REE (kcal/24 h) of subjects at 18°C, 22°C, 28°C and 38°C. Female: Dashed line; Male: Solid line

**Supplementary Figure 2**


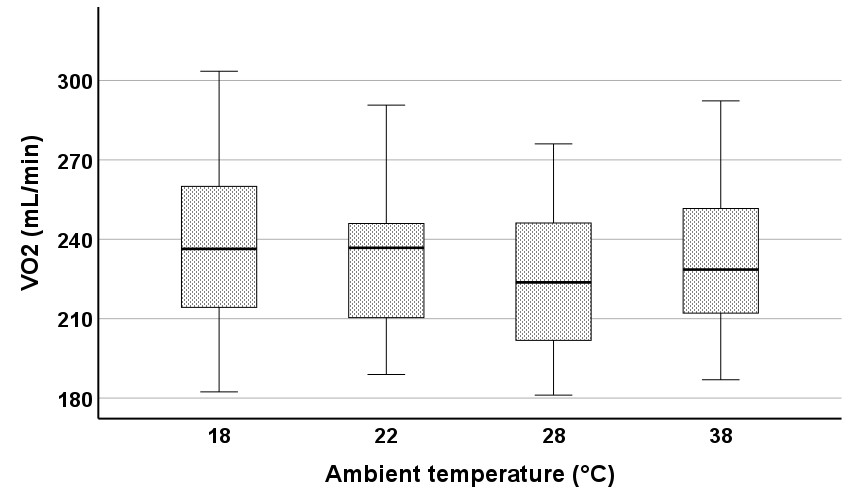


**Supplementary Figure 2:** Boxplots of VO_2_ at different ambient temperatures

**Supplementary Figure 3**


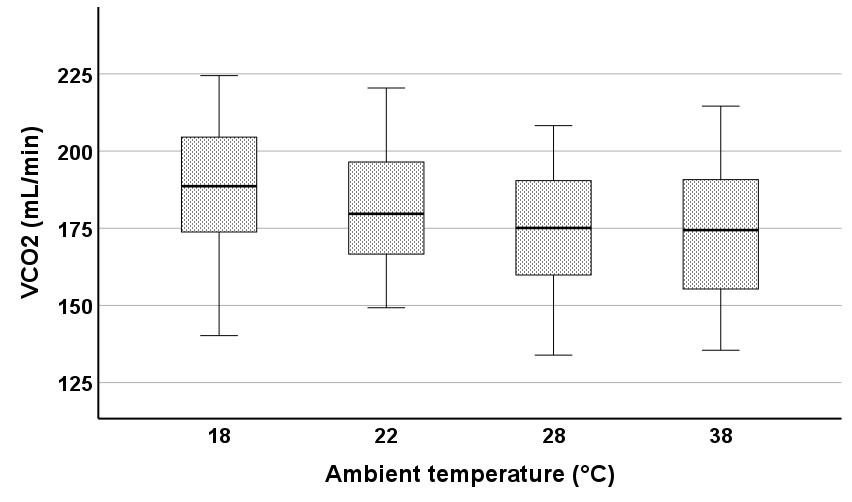


**Supplementary Figure 3:** Boxplots of VCO_2_ at different ambient temperatures
